# Fertility following uterine torsion in dairy cows: A cross-sectional study

**DOI:** 10.14202/vetworld.2020.92-95

**Published:** 2020-01-11

**Authors:** Marlene Sickinger, Eva-Maria Erteld, Axel Wehrend

**Affiliations:** 1 Clinic for Ruminants, Justus-Liebig-University of Giessen, Frankfurter Straße 104, 35392 Giessen, Germany; 2 Clinic for Obstetrics, Gynecology and Andrology of Large and Small Animals with Veterinary Ambulance, Justus-Liebig-University of Giessen, Frankfurter Straße 106, 35392 Giessen, Germany

**Keywords:** dairy cattle, fertility, uterine torsion, uterus involution

## Abstract

**Background and Aim:**

Dairy cows with uterine torsion often are susceptible to reduced fertility resulting in more costs and effort to restore the economy of those cows. The aim of our study was to examine and evaluate the possible associations between uterine torsion and consequent uterine involution disturbances, on the one hand, and between the degree and duration of uterine torsion with fertility parameters, on the other hand.

**Materials and Methods:**

Within 1.5 years, 115 dairy cows (German Browns, German Holsteins, and German Fleckvieh) that were suffering from uterine torsion were examined to evaluate the incidence of involution disturbances of the uterus and to examine the fertility after calving. Statistical analysis included correlation analyses between the degree and duration of torsion and fertility parameters (days open, days to conception, conception rate and services per conception, and intercalving interval) as well as incidence of involution disturbances.

**Results:**

The study revealed no statistically significant correlation between uterine involution and degree of uterine torsion. However, involution processes were significantly correlated to the time of the expulsion of the fetal membranes. Days to conception and intercalving intervals were significantly influenced by the presence of uterine torsion.

**Conclusion:**

Concerning fertility after uterine torsion, it was shown that reduced fertility is associated with the duration of uterine torsion (p=0.02) and time to drop of fetal membranes (p=0.02) but not with the degree of torsion (p=0.27).

## Introduction

Uterine torsion is a well-known cause of dystocia in dairy cattle that often results in the necessity to consult the local veterinarian [1,2]. Torsion of the organ leads to local ischemia, fetal death and may even result in the death of the cow [2].

Published literature mainly deals with the incidence, pathogenesis and therapeutic possibilities, including prognostic parameters. These prognostic parameters are used to evaluate the outcome after uterine torsion for the calves on the one hand and for the cows on the other hand [2-5]. In buffaloes, uterine torsion is also described in detail [6]. According to these authors, uterine torsion is seen in 0.5-1% of all births and represents 2.7-65% of all reasons for dystocia that is presented to the local veterinarian. Pathogenesis still is not completely clear, but several risk factors have been suggested. Among these are age, breed, anatomical reasons, electrolyte imbalances, abrupt movements of the pregnant cow as well as weight and gender of the calf [1].

The therapy of uterine torsion mainly consists of retorsion of the organ and development of the calf. To perform retorsion, several conservative methods as well as laparotomy have been described. The prognosis for the calf and the cow depend on the onset of the disease as well as on the degree of torsion of the organ. Puerperium and the followed fertility are presumed to be influenced by uterine torsion [4].

To the owner, a positive outcome includes a vital calf and a high yielding and fertile dairy cow that gets pregnant by the first service. To realize an immediate onset of the reproductive cycle, the uterine involution and the ovarian function have to be undisturbed [7]. Dystocia, in general, is associated with the retention of fetal membranes [8].

The aim of our study was to examine and evaluate the possible associations between uterine torsion and consequent uterine involution disturbances, on the one hand, and between the degree and duration of uterine torsion with fertility parameters, on the other hand.

## Materials and Methods

### Ethical approval

Examinations and treatments were performed according to the standard therapeutic measures without any unnecessary harm to the animals. Approval from the Institutional Animal Ethics Committee was not required; the study did not affect the animals in excess of therapy.

### Animals and study design

The present study included 115 dairy cows with uterine torsion of different breeds (German Browns, German Holsteins, and German Fleckvieh). All animals were housed on farms in the south-east of Germany. The diagnosis of uterine torsion was confirmed by the local veterinarian through vaginal and transrectal examination. The duration of uterine torsion was defined as time from the detection of stagnant calving to the retorsion of the organ, whereas the degree of torsion was determined during therapy. To evaluate the general condition of the patients, the animals were examined clinically and gynecologically through manual palpation of the vagina and cervix. Therapeutic measures (transcervical retorsion of the uterus or cesarean section) depended on the degree of the cervical opening. To evaluate the postpartum period, the cows were examined on days 10 and 15 after calving. A classification of the general condition as “undisturbed,” “slightly disturbed,” and “severely disturbed” based on the vital parameters and the behavior of the animals. For a description of the postpartum period, the time to the expulsion of the fetal membranes (normal: 12 h), food intake after calving, and milk yield and general condition of the cows were quoted. At days 10 and 15, after calving, a vaginoscopic and manual examination to assess the cervix and vaginal canal as to trauma, color of the mucous membranes, opening of the cervix, and quality of vaginal discharge were performed. Uterine size and filling (if present) were evaluated through transrectal palpation. Based on these examinations, the following involution statuses were classified: Physiological involution of the uterus (uterus transrectally definable, and unremarkable vaginal discharge), slight disturbance of uterine involution (uterus not definable, but tensed, and purulent vaginal discharge), and severe disturbance of uterine involution (uterus not definable and relaxed, and severe watery vaginal discharge).

Fertility assessment included an observation of the cows until they were diagnosed pregnant or until they left the farm. To evaluate fertility, data of artificial insemination including days to the first service (days between calving and first insemination), days to conception (days between calving and 1^st^ day of pregnancy), days between calvings (days from calving to calving), insemination index (number of pregnant cows per number of all inseminated cows), and services per conception (number of inseminations per number of pregnant cows) were documented. Days to the first service and days to conception were compared with a small control group of 3-5 cows of the same farm that was calving within the same time frame. The time between calvings was related to data of the single farms.

### Statistical analysis

Statistical analysis to evaluate the possible statistical differences between cows with and without uterine torsion incorporated the Fisher’s exact test, the Wilcoxon Mann–Whitney test in its exact form, and correlation analyses. All tests were performed with the software package Testimate [9]. For the fertility data days to the first service, days to conception and days between calvings, the one-sided t-test was used. In all tests, results with p<0.05 were regarded as statistically significant.

## Results

**Figure-1:**
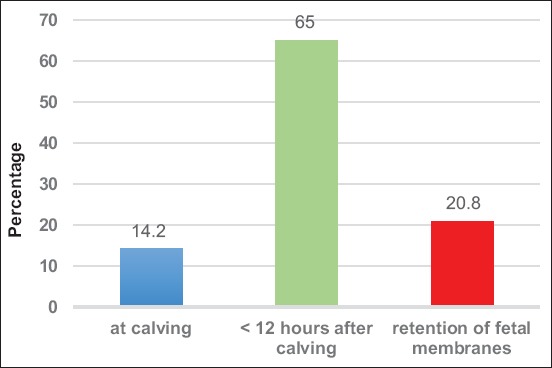
Percentages of dairy cows that showed accelerated, physiological, and delayed expulsion of the fetal membranes.

The present results indicate that the duration of uterine torsion normally was not longer than up to 6 h (49.6 %) with a degree of torsion of 270° (47.7%). The percentages of cows that showed physiological, accelerated, or delayed expulsion of the fetal membranes are shown in Figure-1. Concerning fertility parameters, we could determine an overall pregnancy rate after the uterine torsion of 80.3% with no statistically significant difference in days open. In contrast, the time from calving to the 1^st^ day of pregnancy (days to conception) was significantly prolonged in cows with uterine torsion. Those animals were pregnant 27.6 days later than the healthy controls. Details are given in Table-1. The index of inseminations was recorded as 2.2 and differed not significantly between diseased and healthy animals. In contrast, the time between calvings differed statistically significant between cows with uterine torsion and the healthy controls resulting in a 25.5 days longer time from calving to calving. In this context, the own statistical analyses could show that there is no statistically significant relationship between the degree of uterine torsion and fertility (p=0.27). However, there was a significant correlation between duration of torsion and fertility. With prolonged time of persistent torsion of the uterus, the probability of infertility rose (p=0.02). Infertility also resulted from retention as well as from preterm detachment of the fetal membranes (p<0.0001). Cows with the expulsion of the fetal membranes simultaneously with calving seem to be more prone to infertility than cows suffering from retained fetal membranes (RFM) because neglect of those cases with preterm detachment results in a marked reduction of significance (p=0.05).

**Table-1:** Fertility parameters in cows with and without uterine torsion. p<0.5 indicate significant differences between the groups.

Fertility parameter	Uterine torsion	Healthy cows	p-value
Days open	86.6±42.7	79.2±24.5	Not significant
Days to conception	143.7±74	108.8±36.7	0.013
Intercalving intervals	434.6±74.1	410.1±21.2	0.018

The examination concerning the influence of uterine torsion on involution processes displayed a statistically significant enhancement of expulsions of the fetal membranes simultaneously to calving in correlation with the degree of uterine torsion (p=0.03). In severe cases of uterine torsion (>360°), the fetal membranes always could be removed together with the dead calf. A retention of the fetal membranes, on the other hand, develops independently from the degree of torsion (p=0.83). The examination of the relationship between degree of torsion and involution of the uterus showed no significant correlation between these parameters (p=0.45 with R=0.076). A subsequent disturbance of uterus involution is statistically more probable after retention (p<0.0001) as well as after simultaneous expulsion of the fetal membranes at calving (p=0.0001).

## Discussion

Knowledge concerning the restoration of the reproductive cycle after uterine torsion is extremely guarded and either restricted to the breed Brown Swiss or to a somewhat biased collective of animals within a clinic for obstetrics [3,10]. The own results are the first attempt to examine fertility parameters and the incidence of uterine involution disturbances after uterine torsion by a controlled prospective clinical study including the breeds Brown Swiss, German Holsteins, and German Fleckvieh cows. We compared the results of our evaluation of fertility data with control cows from the same farm. This intra-farm comparison of diseased and control cows enables an unbiased data analysis concerning housing or management-related risk factors for the development of uterine torsion, calving management, and fertility status within the different farms. In doing so, we could show that days to conception and intercalving intervals were significantly influenced by the presence of uterine torsion. The reason for this finding is supposed to relate to uterine tissue damage rather than to a delayed onset of ovarian activity because only days to conception and intercalving intervals but not days open were found to differ between cows with and without uterine torsion.

In general, the postpartum period is of extraordinary importance for undisturbed fertility. One of the main adverse factors resulting in impaired fertility is RFM with consecutive metritis [11]. In the present study, animals suffered from this disease in 20.8% of all examined cases. This observation is in line with a retrospective study in Switzerland in Brown Swiss cows, where an observation incidence for RFM of 21.2% could be shown for this breed. The physiology of placental detachment is a complex, multifactorial system with many influencing factors as to its impairment [11]. The detachment of the fetal membranes may not only be delayed but also in severe cases of uterine torsion may be preterm. According to Schonfelder and Sobiraj [3], such a detachment takes place in 81.8% of all cows undergoing cesarean section for the correction of uterine torsion. The authors suggest that this very high rate of detached fetal membranes may be correlated to the high rate of fetal deaths during torsion of the uterus. The present study revealed a much less ratio of detached fetal membranes with 14.2% of all observations, but we agree with the surveillance that this detachment was related to fetal death. One possible explanation for this incongruity might be that Schonfelder and Sobiraj [3] refer to a collective of clinical cases whereas our own study represents a controlled prospective clinical field study with an unbiased population of animals. In contrast to the assumption, that ischemia of the uterine artery may induce loosening of the cotyledon-caruncle interface [3], other authors have suggested that after uterine torsion a delayed detachment of the fetal membranes occurs [12]. Non-inflammatory edema of this interface because of compression of the venous uterine outflow is proposed as the reason for a delayed loosening of the fetal membranes.

Whereas the involution of the uterus was undisturbed in only 48.4% of all cows in the present study, we diagnosed also a remarkable amount of mild (31.6%) to severe (20%) impairments of uterine involution. The overall ratio of uterine involution disturbances in the present study seems comparatively high with 51.6% but is in accordance with the results of Schonfelder and Sobiraj [3] who could show involution disturbances in 63% of all cases after uterine torsion and cesarean section. Klein and Wehrend [12] explain this postpartum delay of uterus involution by severe uterine tissue damage in cows with torsion uteri. As this tissue damage occurs independently from RFM and because our results present a statistically significant impairment of uterine involution in cows with the preterm detachment of the fetal membranes, this symptom may serve as an in-field prognostic parameter for fertility. In addition, the results from Klein and Wehrend [12] also explain why fertility is reduced after uterine torsion. These researchers could show histologically that in cases of uterine torsion with a degree of torsion of 360° massive edema of the uterine wall with the loosening of the endometrium takes place. Combining the own results with the results of Klein and Wehrend [12] leads to the assumption that fertility impairment after uterine torsion is solely related to the described damage of the uterine wall. This hypothesis is based on the fact that we could show a significantly prolonged time to conception but a not prolonged time to the first service in cows with uterine torsion in comparison to healthy animals from the same farm. This means that the ovarian function seems not affected after uterine torsion in contrast to the integrity of the uterine wall and mucous membranes that are essential for the implantation of the embryo. As a consequence, a 27.6-days longer period to conception in those animals with uterine torsion could be shown. A meta-analysis of the effects of disease on reproduction [13] could show a similar result concerning the general effect of dystocia on reproduction. The authors state that cows after dystocia and RFM need 8 and 11 days more to conception than healthy controls. As the reason for dystocia is not clarified in this study, we suppose that uterine torsion is underrepresented in this meta-analysis resulting in a shorter time of delay to conception or in other words, we think, that the impact of uterine torsion on fertility might be more severe than dystocia because of narrowness of the vagina in calving cows.

## Conclusion

The present study suggests that after uterine torsion, an impairment of uterine involution and reduced fertility was seen. Involution disturbances are associated with unphysiological detachment processes (simultaneously to calving and delay) of the fetal membranes. Probability of infertility is supposed to rise with the duration of uterine torsion and seems to be due to tissue damage of the uterine wall resulting in a prolonged time to conception and time between calvings.

## Authors’ Contributions

AW: Conceptualization. EE: Data curation, investigation and formal analysis: EE; Funding acquisition: none; Investigation: EE; AW: Methodology, project administration and review and editing. MS: Drafted and revised the manuscript All authors read and approved the final manuscript.
